# Going to Extremes of Lung Physiology–Deep Breath-Hold Diving

**DOI:** 10.3389/fphys.2021.710429

**Published:** 2021-07-09

**Authors:** Kay Tetzlaff, Frederic Lemaitre, Christof Burgstahler, Julian A. Luetkens, Lars Eichhorn

**Affiliations:** ^1^Department of Sports Medicine, University Hospital of Tübingen, Tübingen, Germany; ^2^Faculte des Sciences du Sport et de l’Education Physique, Universite de Rouen, Rouen, France; ^3^Department of Radiology, University Hospital Bonn, Bonn, Germany; ^4^Department of Anesthesiology and Intensive Care Medicine, University Hospital Bonn, Bonn, Germany

**Keywords:** breath-hold diving, apnea, glossopharyngeal insufflation, decompression, narcosis, lung

## Abstract

Breath-hold diving involves environmental challenges, such as water immersion, hydrostatic pressure, and asphyxia, that put the respiratory system under stress. While training and inherent individual factors may increase tolerance to these challenges, the limits of human respiratory physiology will be reached quickly during deep breath-hold dives. Nonetheless, world records in deep breath-hold diving of more than 214 m of seawater have considerably exceeded predictions from human physiology. Investigations of elite breath-hold divers and their achievements revised our understanding of possible physiological adaptations in humans and revealed techniques such as glossopharyngeal breathing as being essential to achieve extremes in breath-hold diving performance. These techniques allow elite athletes to increase total lung capacity and minimize residual volume, thereby reducing thoracic squeeze. However, the inability of human lungs to collapse early during descent enables respiratory gas exchange to continue at greater depths, forcing nitrogen (N_2_) out of the alveolar space to dissolve in body tissues. This will increase risk of N_2_ narcosis and decompression stress. Clinical cases of stroke-like syndromes after single deep breath-hold dives point to possible mechanisms of decompression stress, caused by N_2_ entering the vasculature upon ascent from these deep dives. Mechanisms of neurological injury and inert gas narcosis during deep breath-hold dives are still incompletely understood. This review addresses possible hypotheses and elucidates factors that may contribute to pathophysiology of deep freediving accidents. Awareness of the unique challenges to pulmonary physiology at depth is paramount to assess medical risks of deep breath-hold diving.

## Introduction

Breath-hold diving (synonyms: apnea diving, freediving, and skin diving) has been practiced by humans throughout history for leisure and commercial purposes, e.g., harvesting sea food or spearfishing. More recently breath-hold diving also became a competitive sports activity, inspired by the famous competition between Italian freediver *Enzo Maiorca*, who breached the 50 m depth in 1960, and French freediver *Jacques Mayol*, who was the first to reach 100 m in 1976. These achievements also raised interest by the scientific community, as they challenged the pertinent understanding of the depth limits of breath-hold diving. This review aims at elucidating the particular challenges of deep human breath-hold diving to respiratory physiology, and to highlight the serious medical risks associated with competitive freediving to great depths.

## The Diving Response

A major challenge for humans inherent to the aquatic environment is the lack of oxygen, as mammals cannot breathe under water. Thus, oxygen depletion relies on air inhaled into the lungs before breath-holding, and intrinsic oxygen stores bound in blood to hemoglobin, in muscle to myoglobin and in smaller amounts also physically dissolved in plasma. Diving mammals have developed a major physiological adaptation to withstand asphyxia for prolonged dive duration called the diving response. This fundamental mechanism enabling organisms to maintain life during asphyxia has been demonstrated in many terrestrial species and involves both bradycardia and peripheral vasoconstriction. In humans, the diving response is characterized by a synergistic sympathetic and parasympathetic activiation and catecholamine increase (Heusser et al., 2009; Eichhorn et al., 2017), redistribution of blood flow to maintain adequate oxygen supply to hypoxia sensible organs (Joulia et al., 2009; Eichhorn et al., 2018), and reduced heart rate (Perini et al., 2008; Costalat et al., 2021). More recently, active contraction of the spleen has also been attributed to the human diving response without clear relationship.

The human diving response is highly variable and can be modified by physiological as well as emotional factors. Elite breath-hold divers have developed certain adaptions to repeated apnea training, such as greater tolerance to hypoxia and hypercapnia, and a more pronounced diving response. For some factors like the blunted chemosensitivity to hypercapnia a possible genetic predisposition has been discussed while apnea training may modify these or other physiologic and psychophysical factors (Bain et al., 2018).

Trained freedivers achieve average breath-hold times of 5 min under resting conditions (Hansel et al., 2009), allowing them in theory to reach great depths with assisted descent using a ballast, and ascending with lifting bags. In fact, total dive time for documented world record dives were less than 5 min. Maximum achievable breath hold time is not determining the maximal diving depth, but physiological factors (lung size and compliance, cardiac fitness), blood redistribution, economization of diving movements, equalization techniques, mental strength and other factors influence the risk for serious hypoxemia upon ascent and are therefore affecting the maximum diving depth.

## The RV/TLC Ratio

It had been generally believed that the maximum depth to which a breath-hold diver could descend would be determined by the individual ratio of residual volume (RV), i.e., the lung volume remaining in the lungs after complete exhalation, to total lung capacity (TLC) (Carey et al., 1956; Agostoni, 1965). As intrathoracic gas will be compressed during a breath-hold dive with increasing depth, volume halves at 10 m depth (2 atm absolute pressure), and will be reduced to one third of its initial volume at 20 m depth. Thus, in a subject with an RV of 20% of TLC, the critical lung volume will be reached at 40 m depth (5 atm absolute pressure) when the TLC is compressed to one fifth of its initial volume. Intrathoracic pressure will become lower than ambient pressure and therefore pressure of body tissues and blood. With further descent beyond RV the distensibility limit of the blood-containing structures in the chest may be reached, resulting in possible fluid extravasation and pulmonary trauma including alveolar damage and intra-alveolar bleeding.

The classical concept that the RV/TLC ratio determined the maximum breath-holding diving depth had been consistent with early research in professional Japanese and Korean diving women harvesting seafood in the Sea of Japan and Yellow Sea. Teruoka (1932) was the first to extensively study the so called Japanese “Ama” women divers and documented maximum diving depths of up to 25 m, although anecdotally reported dives were up to 45 m. A study in US Navy submarine escape training tank instructors reported larger TLC and vital capacity compared to laboratory control personnel, supporting the concept that a lower RV/TLC ratio was beneficial to achieve greater freediving depths (Carey et al., 1956). Divers had 14.6% higher than predicted vital capacity; a longitudinal follow-up over 1 year attributed the higher VC to training adaptation rather than a selection effect. Song et al. (1963) measured RV and TLC in Korean diving woman and controls and determined an average ratio of 25% that allowed dives to approx. 30 m. There was no significant difference between divers and controls, however, diving women tended to have a lower RV/TLC ratio per corresponding age group.

Subsequent reports confirmed that subjects achieving record depths had favorable individual RV/TLC ratios, allowing them to go deeper than predicted. US Navy diver *Robert Croft* had a TLC of 9.1 L and an RV of 1.3 L providing a ratio that would enable him to dive safely up to 60 m depth (Schaefer et al., 1968). In fact, the predicted values according to his age, height and sex would have amounted to 6.9 and 2 L for TLC and RV, respectively, allowing him to hardly reach 30 m. Thus, favorably low RV/TLC ratios enabled certain individuals to reach record depths that exceeded the depth range of 30–40 m known from professional breath-hold divers.

However, *Robert Croft* reached 66 m depth in 1968 and French freediver *Jacques Mayol* became the first to break the 70 m limit in the same year. These achievements alerted physiologists that the concept of RV/TLC determining maximum breath-hold diving depth needed to be refined.

## Thoracic Blood Shift

Although the anatomy of the human lung, i.e., size and properties of lung parenchyma and surrounding organs and tissues, will primarily determine individual gas volumes, physiologic factors such as the force exerted by respiratory muscles, lung reflexes, properties of airways etc. will affect volume changes. RV of the lungs varies markedly with pressure, being greater at positive pressures and smaller at negative pressures (Rahn et al., 1946). Seminal work by Fenn et al. (1947) showed that an increase of pulmonary pressure of 30 cm H_2_O could displace 500 mL or about half of the blood contained in the lungs. Conversely, submersion of the chest reduces lung volumes, equaling an effect of negative pressure breathing (Hong et al., 1960). It was shown that the change in lung volume during submersion was mainly due to the abdomen with the diaphragm displaced cranially (Agostoni et al., 1966). Measuring the diffusion capacity of the lungs for carbon monoxide showed a marked increase during immersion attributable directly to storage in the lung of blood displaced by pressure from the vessels of immersed parts of the body (Guyatt et al., 1965).

Schaefer et al. (1968) measured thoracic blood volume displacements at various depths during breath-hold dives by use of impedance plethysmography. At 30 m depth in the US Navy escape training tank they could show that 1047 mL of blood were shifted into the thorax. Thus, a significant amount of blood shift due to body immersion will replace intrathoracic air and consequently reduce RV. The authors reported that record freediver *Jacques Mayol* who reached 70 m depth had an RV of 1.88 L and a TLC of 7.22 L that would have allowed him to dive to 28 m only. It could be calculated that a blood shift of 980 mL into the thorax was necessary in order to not suffer lung squeeze at 70 m.

The authors also determined oxygen costs of breath-hold dives, showing that oxygen requirement was lower than oxygen consumption during exercise and neither hypoxia nor hypercapnia determined dive limits during these breath-hold dives. The authors had measured gas tensions of mixed expired and alveolar air at various depths during descent and ascent of breath-hold dives (Schaefer and Carey, 1963). Their results indicated that at depth carbon dioxide is transferred back from lung alveoli into the pulmonary capillaries and oxygen (O_2_) content of the lungs does not change linearly, with decreased O_2_ transfer at depth and steep decrease during ascent.

Craig (1968) postulated that during descent the size of the thoracic volume would change with compression of the thoracic cage and elevation of the diaphragm but that this mechanism alone could not account for the depths reached. As the intrathoracic pressure at depth would become less than ambient pressure, there would be a pressure gradient along which blood would shift into the thorax. In an interesting experiment he measured thoracic pressure by esophageal balloon during a breath-hold dive to 4.75 m which the subject started at surface exhaling to RV of 2 L, confirming that 600 mL of blood must have shifted into the thorax to compensate for the compression to 1.4 L. The author speculated “that the capacity of a diver to compress the air volume by fluid changes might be the major limiting factor in the depth of a dive. If as much as 1 L of blood could move from the peripheral to the central circulation, a diver might go as deep as 140 m”.

While these studies elucidated thoracic blood shift during (deep) breath-hold diving as an important physiologic mechanism to prevent lung squeeze, athletes continued to compete for ever increasing record depths (Sharp, 2003). *Umberto Pelizzari* reached 150 m depth in 1999 and *Loic Leferme* set a record depth at 162 m in 2002^1^. These depths could only be achieved using technical assistance such as weight sleds upon descent and balloons to assist ascent from depth, in order to keep breath-hold time to a tolerable minimum. However, consideration of RV/TLC ratio and thoracic blood shift alone would not allow subjects to survive exposures to these depths. An increased awareness by the scientific community of the record depths achieved by some elite breath-hold divers prompted early case studies of elite athletes who used a unique breathing technique to further increase their TLC (Örnhagen et al., 1998; Muth et al., 2003; Simpson et al., 2003; Lemaitre et al., 2010).

## Glossopharyngeal Breathing

Glossopharyngeal breathing was first described as a method of independent breathing in patients with post-poliomyelitic syndrome (Collier et al., 1955). In these patients with pronounced impairment of the respiratory muscles and a consecutively low vital capacity, the technique allowed an increase in VC of 5.4 times, thereby prolonging breathing time without mechanical aid by 184 times. With technical progress in ventilation the technique got somewhat forgotten, however, more recently was recognized as a method used by elite breath-hold divers to improve performance. Increasing the volume of intrathoracic air will further reduce the RV/TLC ratio, thus, allowing athletes to dive deeper before reaching the critical lung compression point for lung squeeze. Moreover, it will increase pulmonary oxygen stores and thereby breath-hold duration, and dispose of greater air volume to equalize cranial air cavities (ears, sinuses) at depth. It must be acknowledged that TLC is not equivalent to maximum lung volume, and due to its enormous distensibility the lung can stretch to above normal volumes.

Glossopharyngeal insufflation is started after filling the lungs to TLC. Then a mouthful of air with the glottis closed is compressed by the oropharyngeal muscles and then forced into the lungs. Before the pharyngeal muscles are relaxed the glottis is closed to allow new air entering the oral cavity. This buccal pumping cycle is repeated several times until a sensation of fullness occurs (see Supplementary Video). By applying this technique, elite breath-hold divers may increase their TLC by up to 47% (Loring et al., 2007); mean increases in TLC were reported in several larger samples of elite breath-hold divers to average 15–25% (Lindhom and Nyren, 2005; Overgaard et al., 2006; Seccombe et al., 2006; Tetzlaff et al., 2008). The number of buccal pumping cycles employed to reach the individual sensation of fullness varies greatly across individuals; a median of 19 (range 13–75) were reported by Tetzlaff et al. (2008).

Seccombe et al. (2006) recorded mouth relaxation pressure immediately and 5 min after glossopharyngeal insufflation and plotted this vs. plethysmographic volume change. They calculated that the mean increase in TLC attributable to overdistension of the lung was 69%, while the remainder was attributable to air compression. Since glossopharyngeal insufflation must overcome lung recoil pressure to increase lung volume above TLC, the authors speculated that recoil pressure at high lung volumes may be altered transiently, supported by the fact that TLC 5 min after glossopharyngeal insufflation still was above expected normal variation. Indeed, static lung compliance was measured during and up to 3 min after glossopharyngeal insufflation in five elite breath-hold divers (Tetzlaff et al., 2008). A transient decline of lung elastic recoil could be confirmed as compliance was still elevated when subjects had exhaled and returned to tidal breathing.

**FIGURE 1:**
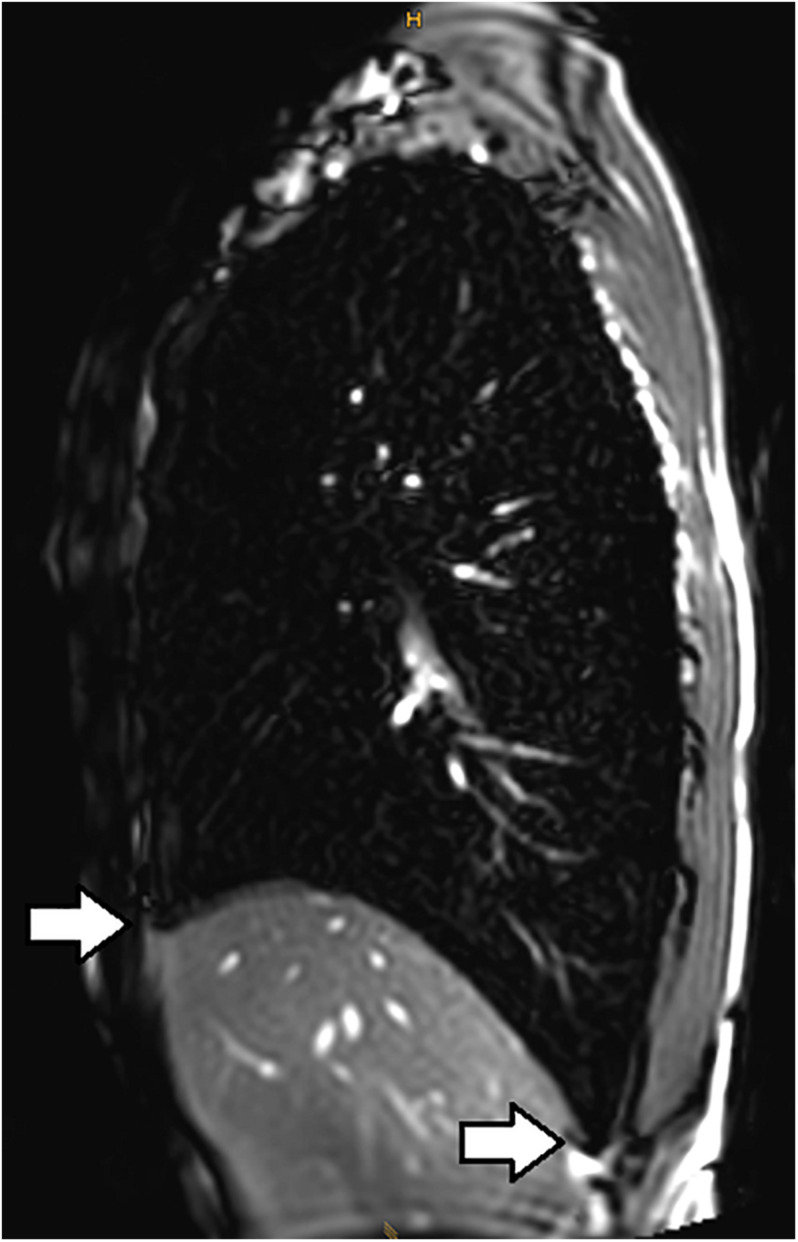
Subxiphoidal and costodiaphragmatic recessus (marked with white arrows) filled with air during glossopharyngeal insufflation.

**FIGURE 2:**
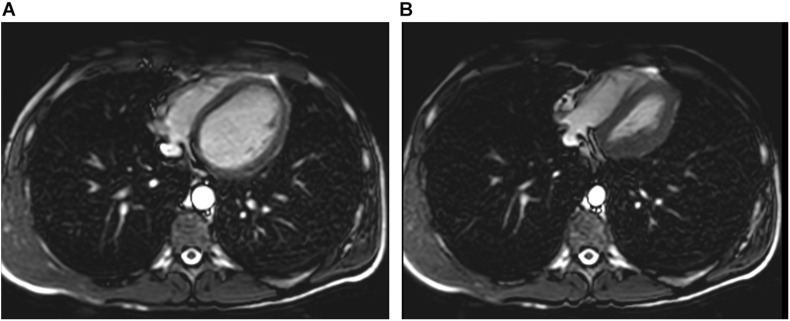
Pulmonary hyperinflation disrupts cardiac filling and leads to cardiac dysfunction. Panel **(A)** axial slice shows normal filling whereas Panel **(B)** demonstrates cardiac dysfunction with impaired left ventricular filling and right ventricular overload (D-shaped configuration) and reduced aortic diameter.

Lindhom and Nyren (2005) also investigated magnetic resonance imaging (MRI) during glossopharyngeal insufflation and described a reduction of thoracic blood volume with emptying of the vessels and heart, and a downward shift of the diaphragm. In a study employing dynamic MRI and spirometry, Eichinger et al. (2008) demonstrated that the shape of the thorax was primarily preserved despite the marked increase in total lung volume as measured by MR-spirometry. Herniation of the lung underneath the sternum and enlargement of the costodiaphragmatic angle furthermore demonstrated the distensibility and high performance of trained lungs (see Figure 1). Seccombe et al. (2010) measured single photon emission computed tomography (CT) of the chest after labeled albumin injection and showed markedly diminished or even absent lung perfusion in areas of expanded lung. Lung hyperinflation induced by glossopharyngeal insufflation elicits significant hemodynamic effects, including a decrease in systemic arterial blood pressure as well as decrease in cardiac output (Potkin et al., 2007). Hypotension with glossopharyngeal breathing is associated with acute biventricular systolic dysfunction (see Figure 2, video axial sequence 113–293). Cardiac MRI revealed changes resembling pulmonary arterial hypertension which however were reversible shortly after cessation of voluntary lung hyperinflation (Eichinger et al., 2010). Quantitative analyses of pulmonary and central blood volume changes revealed an almost 50% decrease of central blood volume after lung hyperinflation in the non-immersed state (Mijacika et al., 2017a), and it could be shown that this lung hyperinflation results in translocation of blood from the central blood volume into abdominal, pelvic, and peripheral veins (Mijacika et al., 2017b).

Obviously, glossopharyngeal insufflation exerts considerable mechanical stress on lung elastic properties. Increases in intrapulmonary and transpulmonary pressures up to 109 cmH_2_O and 80 cmH_2_O, respectively, have been measured after glossopharyngeal insufflation (Loring et al., 2007). These data indicate that some individuals can withstand transpulmonary pressures and volumes far greater than those to which lungs would normally be exposed to. However, in one subject the authors reported evidence of asymptomatic mediastinal emphysema by CT of the chest (Jacobson et al., 2006), suggesting that forceful glossopharyngeal insufflation at volumes above TLC can lead to lung rupture with air dissecting centrally along the vessels into the mediastinum. Chung et al. (2010) subsequently studied glossopharyngeal insufflation in a sample of six elite breath-hold divers who performed routine maneuvers with CT scans obtained after a maximal glossopharyngeal breathing maneuver and a control CT scan at least 3 days later at TLC. A pneumomediastinum was revealed in five subjects that were able to increase thoracic gas volume above TLC. In two subjects mediastinal air was still present after 3 days although the total amount was reduced. None of the subjects at any time reported symptoms of chest discomfort, dyspnea, neck pain, or difficulty swallowing or voice change. Based on these results, the authors speculated whether glossopharyngeal insufflation may lead to long-term damage to the lungs.

Pulmonary function testing of elite breath-hold divers consistently showed higher vital capacity than predicted from population derived equations (Overgaard et al., 2006; Tetzlaff et al., 2008; Lemaitre et al., 2010). Elite breath-hold divers consistently had higher VC compared to age-matched controls (Tetzlaff et al., 2008; Lemaitre et al., 2010). There is only scarce information on longitudinal lung function changes with continuously practiced glossopharyngeal breathing. Walterspacher et al. (2011) reported an increase in ventilatory volumes and normal compliance in four elite breath-hold divers that had been available for a 3-year follow-up. At that time the mean breath-hold diving performance in the subjects amounted to 6.6 years, indicating that the stress on the pulmonary parenchyma imposed by glossopharyngeal insufflation may be transient and reversible. Seccombe et al. (2013) interpreted an increase in VC, TLC, and functional residual capacity in a breath-hold diver over 8 years as a reduction of chest wall recoil at higher lung volumes, in consequence of repeated glossopharyngeal insufflation. These results should be interpreted with caution due to small sample size and only limited follow-up. However, the follow-up supports the cross-sectional findings of large vital capacity, probably due to training due to structural adaptation to repeated lung extension.

Lindhom and Nyren (2005) also reported a breathing method similar to glossopharyngeal insufflation but reversing the pumping maneuver, called glossopharyngeal exsufflation. The procedure is used to by breath-hold divers to suck air into the mouth at great depth as respiratory muscles will not be able to provide the force needed against the pressure difference. This air will be needed to equalize middle ear pressure when going deeper. The method is also employed for training purposes as divers can simulate greater depth when exhaling beyond RV and diving in a swimming pool. Lindhom and Nyren (2005) reported that RV could be reduced by 21%. Loring et al. (2007) confirmed this magnitude of reduction in RV and measured intrapulmonary pressures of −10 to −30 cmH_2_O, probably indicating the point of complete airway closure. The authors used MRI lung imaging with inhaled hyperpolarized ^129^Xe to characterize lung inflation from sub-RV volumes after glossopharyngeal exsufflation (Muradyan et al., 2010). MRI demonstrated sharply demarcated ventilated vs. non-ventilated regions of the lungs of the divers, indicating punctate opening, secondary to frank airway closure during the preceding glossopharyngeal exsufflation used to reach those low volumes.

Is there a limit to deep breath-hold diving?

Summarizing the above, it becomes obvious that prior physiologic concepts for the depth limits of breath-hold diving have been refuted. The RV/TLC ratio is an important determinant of the depth a breath-hold diver may descend to without harm, but thoracic blood shift, and individual adaptations such as glossopharyngeal insufflation and exsufflation allow to reach considerably greater depths than previously assumed. For example, an elite breath-hold diver with an RV of 1.7 L and TLC of 10 L (measured) would be able to descend to 49 m depth according to RV/TLC ratio. With thoracic blood shift of 1000 mL (assumed) the depth limit would be at 134 m, because RV would be reduced to 0.7 L. Increasing TLC with glossopharyngeal insufflation to 13.2 L (measured) he could go to 179 m, and with glossopharyngeal exsufflation of 0.4 L (measured) maximal depth would be at 320 m (personal communication, Dr. C.M. Muth, Ulm, Germany). Accordingly, there had been a fast evolution of depth records with ever increasing depths up to the beginning of the new millenium (Ferretti, 2001). Austrian freediver *Herbert Nitsch* set the official world record at 214 m depth in 2007^2^. Thus, pulmonary anatomy and physiology could not be accounted to limit the breath-hold diving depths achieved by competitive athletes.

*Herbert Nitsch* tried to surpass his own record in 2012 and reached a depth of 244 m; however, when surfacing he presented with a stroke-like clinical syndrome with loss of consciousness and needed immediate medical support. Permanent disability caused him to end his career. His medical condition was attributed to decompression illness.

## Decompression Stress

Another area where previous concepts of physiology needed to be revised more recently is the possibility of decompression stress in breath-hold diving. Historically it had been widely believed that diving mammals and human freedivers alike were immune to decompression illness, since nitrogen (N_2_) uptake from alveolar air is restricted to the available amount of gas in the lung at depth (Lanphier, 1965; Kooyman and Ponganis, 1997). Marine mammals dive routinely and repeatedly to impressive depths without obvious injury, and the only inert gas added is the N_2_ that remains in the lungs from the inhalation before submerging. Studies have suggested that behavioral and physiological adaptations such as the reduction in blood flow to non-essential tissues and a progressive collapse of alveoli would prevent them from N_2_ uptake and thereby minimize decompression stress. However, recent postmortem studies in stranded beaked whales and sperm whales pointed to lesions consistent with acute trauma due to *in vivo* bubble formation resulting from rapid decompression and adverse long-term sequelae, respectively, in these species (Jepson et al., 2003; Moore and Early, 2004). These recent observations led to the understanding that diving mammals are in fact prone to decompression stress during ascent. They obviously manage their inert gas load by physiological responses that are still poorly understood, yet in certain situations may suffer decompression illness if they ascend from depth too quickly, e.g., by irritation through sonar noise (Hooker et al., 2012).

An important physiological adaptation of diving mammals to minimize decompression stress is lung collapse. The early occurrence of lung collapse at relative shallow depths of around 50 m of seawater will entirely curtail any gas exchange between the lungs and the blood and, thus, restrict N_2_ absorption during the dive. In contrast, the human chest and lungs are much less compressible. Human airway and alveolar compression and re-expansion during deep breath-hold dives were studied using a computational model of the human respiratory tract (Fitz-Clarke, 2007). The model predicted total lung collapse with degassing of all alveoli at a depth of approximately 235 m, which is considerably deeper compared to the collapse depth in diving mammals. It has also been calculated that human lungs are highly efficient at gas exchange just prior to total collapse (Fitz-Clarke, 2009) and N_2_ uptake will increase linearly during descent up to that point. Thus, in humans, there will still be N_2_ absorption at greater depths, and with glossopharyngeal insufflation significantly more N_2_ will be available for uptake.

The evidence that decompression sickness from repetitive breath-hold diving may occur in humans has meanwhile been established (Schipke et al., 2006; Lemaitre et al., 2009; Schipke et al., 2019). Apart from clinical pathology findings there has also been proof of intravascular gas bubbles by Doppler measurements in professional Japanese breath-hold divers after a series of repetitive dives to depths of 20 m (Lemaitre et al., 2014), and tear film bubble formation has been reported in submarine escape tank divers after repeated breath-hold dives to 30 m (Sheard, 2008). However, it has been considered unlikely that breath-hold divers would be submersed for sufficient time and sufficient depth to suffer decompression stress after single deep dives.

There is increasing evidence in humans that decompression stress may apply to single deep breath-hold dives: Schagatay (2011) reported cases of repeated breath-hold dives to 45 m depth presenting with paralysis of the limbs, and a case of speech disturbance and amnesia after a final single free dive to 90 m. In an ultrasound study after single deep dives up to 70 m there were a couple of subjects with confirmed low grade gas bubbles in cardiac chambers. Finally, an increasing number of serious incidents during deep breath-hold dives indicated the possibility of neurological insult, e.g., stroke, due to cerebral arterial gas embolism as a consequence of decompression stress (Tetzlaff et al., 2017). Remarkably, these cases occurred in young healthy subjects without a history of cardiovascular disease or other pertinent risk factors.

## Pulmonary Arteriovenous Shunts

Usually the capillary bed of the pulmonary vasculature is an effective filter for venous N_2_ microbubbles (Butler and Hills, 1985). However, bubbles may bypass this filter through a right-to-left shunt, e.g., patent foramen ovale. The presence of a persistent patent foramen ovale has indeed been associated with the risk of decompression illness in self-contained underwater breathing apparatus (SCUBA) divers (Wilmshurst et al., 1989). However, arterial gas bubbles have been reported in SCUBA divers without a patent foramen, indicating the possibility of microbubble transfer through extracardiac shunts (Gerriets et al., 2000). In fact, intrapulmonary arteriovenous anastomoses may offer another opportunity for venous gas bubbles to bypass the capillary filter and become arterialized (Madden et al., 2015; Schipke and Tetzlaff, 2016). The existence of these anastomoses in human lungs has been shown in morphological studies (Tobin, 1966).

Several factors may facilitate the opening of intrapulmonary shunts, including body positioning (Stickland et al., 2004), physical exercise (Eldridge et al., 2004), and hypoxia (Lovering et al., 2008). It appears unlikely that body position or exercise would be relevant factors during deep breath-hold dives where the diver is using a weighted sled to descend down a rope, and ascend with lifting bags. Hypoxia, however, is increasing with breath-hold duration and alveolar pO_2_ may reach values as low as 30 mmHg after surfacing from deep breath-hold dives (Ferretti et al., 1991). Thus, hypoxia may allow transpulmonary passage of venous N_2_ microbubbles, although it is yet unclear whether acute severe hyperbaric hypoxia would have the same effect on intrapulmonary arteriovenous anastomoses as prolonged normobaric hypoxia. As the N_2_ gas bubble will increase in size during ascent and probably coalesce with other bubbles, greater sized bubbles may be present on the outflow side than what originated on the inflow, and hence not be restricted by the diameter of the shunting vessel (Kerut et al., 2014). As airway closure during atelectasis or lung collapse prevents the ventilation of alveoli supplied by that airway, there will be right-to-left shunt of blood perfusing those alveoli. However, this would require the existence of venous N_2_ microbubbles during the descent of the dive already which is unlikely to occur. As gas exchange in the human lung is still occurring at greater depths, venous gas bubbles may be formed along the extreme N_2_ gradient between tissues and the alveolar space, and eventually these bubbles may arterialize through intrapulmonary arteriovenous anastomoses that are opening during ascent from a deep breath-hold dive triggered by severe hypoxia.

## Nitrogen Narcosis: Myth or Reality?

Inert gas narcosis (IGN) is a well-described phenomenon of subjective and objective mental disturbance due to the narcotic effects of certain inert gases at increased pressure. In SCUBA diving, IGN is mainly related to nitrogen (Clark, 2015). One of the first reports of what is now known as nitrogen narcosis was by *Colladon*, a French physician who, in 1826, descended to 20 m in a diving bell. He described “a state of excitement as if I had drunk some alcoholic liquor” (Unsworth, 1966). In 1935, Behnke first suggested that nitrogen gas might have been the mediator of the observed behavior, by utilizing different breathing gas mixtures in their experiments (Behnke et al., 1935; Grover and Grover, 2014). Nitrogen narcosis can impede cognitive functions and physical performance from depths as low as 10 m, and will be apparent around 30–40 m depth (Rostain and Lavoute, 2016). Symptoms include spatial and temporal disorientation, memory impairments, euphoria, hallucinations, mood changes, impaired neuromuscular coordination, psychomotor and intellectual decrements and unconsciousness (Rostain et al., 2011; Rostain and Lavoute, 2016; Jain, 2017). Loss of consciousness occurs at pressures higher than 11 ATA (Rostain and Lavoute, 2016). These symptoms may affect diver’s safety under water, and contribute directly to up to 6% of deaths in SCUBA divers and/or can be indirectly associated with other diving incidents at depth (Clark, 2015).

During deep breath-hold dives, elite freedivers quite often describe narcosis-like symptoms (personal observation and testimonials from freediving champions *Guignes*, *Zucchari*, *Boudhiaf*, *Streeter*, and *Néry*). Among the symptoms regularly reported by freedivers are hallucinations, the sensation of dreaming or being in a dream, altered perception of time, the sensation of numbness in the mouth and tingling in the fingers and toes, blurred vision, loss of memory, resulting in confusion and panic. The confusion is such that some freedivers practising No-Limit freediving (i.e., assisted freediving with a sled) say they can’t remember what they did on the bottom and on part of their ascent from depth. Schagatay (2011) reported that among 24 divers during a competition, 12 experienced symptoms that could be associated with narcosis including dizziness and confusion, and these were all evident at depths greater than 40 m. Others freedivers report depths ranging from 50 to 60 m during training “shallow” dives with hangs at the bottom. The symptoms therefore seem to be quite similar to those of SCUBA divers. However, one of the main differences between SCUBA diving and freediving is the onset of narcosis. While it ususually occurs during the descent of a SCUBA diver using air as breathing gas, it occurs mainly on the way up and sometimes all the way to the surface in freedivers (personal observation and observation reported from *Guillaume Néry*). Thus, it appears that presentation of narcosis in breath-hold diving is distinct from SCUBA diving, and mechanisms of narcosis may be altered.

While the exact mechanisms of IGN remain to be poorly understood, biochemical theories based on gas toxicity and dissolution are the assumptions generally reported for air diving. The anesthetic potency of a narcotic gas has been commonly found to be correlated with the lipid solubility (Meyer-Overton theory). Thus, the site of the IGN is on a lipophilic (fat-soluble) portion of the cell membrane. Due to their lipophilicity, N_2_ and other inert gases bind to membranes. This can cause the membrane to swell beyond a certain critical volume and cause narcotic effects (Rostain et al., 2011; Clark, 2015; Rostain and Lavoute, 2016) with an individual susceptibility, i.e., concentration threshold. Since pressure affects narcotic potency in a linear fashion and nitrogen narcosis does not disappear with decreasing pressure (during ascent) in freediving, it seems that the effects of IGN are more complex in the freediver.

In contrast to SCUBA diving, the intrapulmonary gas of the freediver is not renewed since he is apneic throughout the dive. Thus, the containing air and its composite gases (nitrogen, oxygen, and carbon dioxide) are subject to the effects of (substantial) environmental pressure change while being consumed (oxygen, O_2_) or produced (carbon dioxide, CO_2_) during apnea. On descent, the alveolar and arterial pressures of oxygen increase and can lead to transient hyperoxia at the bottom (Bosco et al., 2018). While the arterial pressure of O_2_ (PaO_2_) can increase during descent, the arterial pressure of CO_2_ (PaCO_2_) remains almost stable because of its markedly greater solubility in blood and tissue and the buffer capacity of the human body for CO_2_. During the descent hypercapnia does not appear to be present even during a constant weight dive to 60 m. Also in case of constant weight apnea (therefore with muscular effort) or assisted apnea (with a sled for example), hypercapnia seems moderate or even non-existent on ascent (Bosco et al., 2018; Scott et al., 2021); thus, it’s contribution to the narcotic effect seems negligible. Due to the complex interaction of gases, activities, and environmental conditions during a dive, nitrogen, oxygen and carbon dioxide may modify the cognitive performance for different partial pressures. It is therefore possible that there is an interplay between O_2_ and CO_2_ that can increase narcosis. However, these data have been established for divers with equipment breathing different gas mixtures (Freiberger et al., 2016; Rocco et al., 2019); no data are available from breath-hold diving where hypercapnia and hypoxia are prevailing. Since PaO_2_ increases during descent there is transient hyperoxia, however, this does not seem to generate oxidative stress in the freediver (Joulia et al., 2003). Conversely, animal studies in rats showed, that hypercapnic hyperoxia stimulates reactive oxygen species (ROS) production in the caudal solitary complex of rat brain but does not induce oxidative stress (Ciarlone and Dean, 2016a). This is probably due to antioxidant and proteosomal removal of damaged lipids and proteins to maintain cell viability and avoid death during protracted hyperoxia (Ciarlone and Dean, 2016b). After a dive, overproduction of ROS and consequent oxidative damage to lipids of membrane and antioxidant capacity decreasing may therefore reflect a occcurrence of hypoxic conditions (Mrakic-Sposta et al., 2019). More studies are needed to determine the impact of ROS-producition on free-divers. On ascent, total pressure decreases, which can accentuate the drop in O_2_ in the blood during muscular effort to dramatic levels. The freedivers become hypoxic to the point of unconsciousness (Muth et al., 2003; Lindholm and Lundgren, 2009; Costalat et al., 2017). Even in the absence of symptoms, these repeated hypoxias seem to impact the cognitive functions of freedivers. Billaut et al. (2018) showed that trained freedivers had alterations in episodic memory in relation to their level of training and the number of syncopes. Finally, several studies show that these hypoxias may have deleterious effects on the brain health of freedivers, although it is not yet possible to assess the long-term consequences (Billaut et al., 2018).

As described above, a certain analogy exists between the narcotic effect of nitrogen in deep diving and the pharmacokinetics of conventional anesthetic gases. The physiology of anesthetic gases has been adequately studied (Kety, 1950; Tanner, 1982), and modern computer programs can predict the uptake and onset of action of anesthetic gases with reasonable accuracy (Athiraman et al., 2016). During induction of narcosis, high partial pressures of anesthetic gases are inhaled to achieve a fast flooding at the target organ. The anesthetic gases dissolved in the blood diffuse to their site of action driven by the concentration gradient. After the induction-phase, the inspiratory gas fraction of the anesthetics can be significantly reduced because the partial pressure in the tissue is approximately equal to the partial pressure in the blood. The speed of partial pressure changes in the different compounds depends, amongst other factors, on cardiac output (Munson et al., 1973), the solubility coefficient and the type of anesthetic gas. If the gas partial pressure is reduced at the end of surgery, the effect of the anesthetic gas persists until the tissue partial pressure at the target organ has dropped. Thus, there is a time delay in both the initial flooding and the descent of the gas. Similar time-course effects may apply to narcotic effects in deep breath-hold diving. During the diver’s descent there is an increase in the N_2_ partial pressure due to the elevation in the ambient pressure. Since the apnea dive generally lasts only a few minutes, only a relatively short time window is available for the saturation phase. The temporal latency of the narcotic nitrogen effects, which coincides with the ascent process, could therefore also be explained by delayed tissue kinetics.

Lastly, there are possible implications of the diving reflex in breath-hold diving narcosis. Apnea induces a reflex mechanism known as the diving reflex, which is mainly characterized by bradycardia and peripheral vasoconstriction that allows blood to be redirected to oxygen-dependent organs such as the heart and brain (Joulia et al., 2009; Lindholm and Lundgren, 2009; Dujic and Breskovic, 2012; Eichhorn et al., 2017; Bain et al., 2018; Elia et al., 2021). This mechanism is more or less present depending on the intensity of the effort, but it seems to be persistent during ascent of constant weight diving (Lemaître et al., 2013). Conversely, this vasoconstriction progressively leads to increases in mean arterial pressure (Breskovic et al., 2011). We have just shown that at the end of a deep apnea, the freediver is hypercapnic, hypoxic, and hypertensive with a significant increase in cerebral blood flow (Joulia et al., 2009; Bain et al., 2018; Eichhorn et al., 2018). Hypoxia, but especially hypercapnia, increases this cerebral blood flow, which can disrupt neuronal activity or at least dynamic brain autoregulation. Indeed, it has also been shown that, in elite freedivers, dynamic brain autoregulation can be severely impaired during maximal apneas (Moir et al., 2019), and that cerebral oxidative metabolism can be decreased. A disruption of the blood–brain barrier has even been suggested (Bain et al., 2018). It may be speculated that the mechanism of central blood shift during descent probably reverses on ascent although this has not been investigated in detail. The diving reflex may contribute to maintained cerebral blood flow during ascent which can be increased by diaphragmatic contractions (Fagoni et al., 2017). Of note, also speed of descent and ascent may influence the risk of syncope (Poiret et al., 2020). Indeed, in constant weight divers using fins, divers with a syncope had longer dive times (197 s vs. 167 s) with a faster first diving phase (the active one) (1.24 m.s^–1^ vs. 1.06 m.s^–1^). It is therefore possible that this higher speed leads to a greater consumption of air from the beginning of the apnea, which will have the effect of reducing the freediver lung volume, accentuating his blood shift and further increasing his cerebral blood flow throughout the dive. These mechanisms all together may contribute to disturbances of cerebral blood flow during ascent, which may impair neuronal activity and cause signs of narcosis experienced by freedivers.

## Conclusion

**FIGURE 3:**
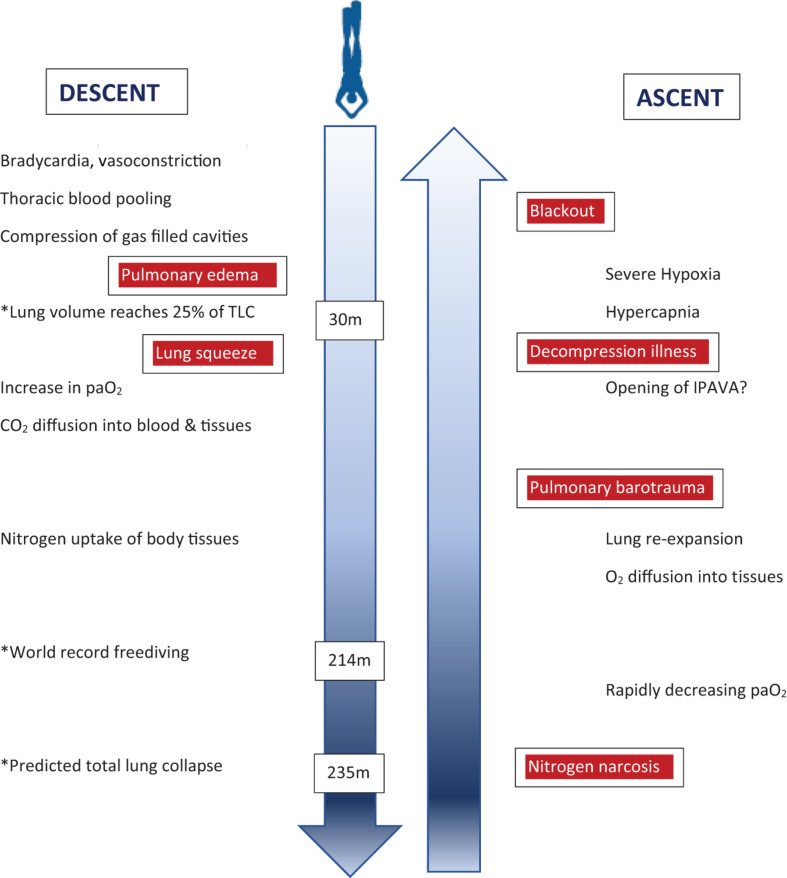
Illustration of physiological changes and health risks (boxed red) during the descent and ascent of a deep breath-hold dive. Some examplary depths are marked (^∗^) to indicate critical physiological challenges or records achieved. Of note, health risks are not related to certain depths but rather depth ranges during descent or ascent, respectively. paO_2_ = arterial oxygen pressure; IPAVA = intrapulmonary arteriovenous anastomoses.

Previous physiologic concepts for the depth limits of breath-hold diving have been refuted more recently. The RV/TLC ratio is an important determinant of the depth a breath-hold diver may descend to without harm, but thoracic blood shift, and individual adaptations such as glossopharyngeal insufflation and exsufflation allow to reach considerably greater depths than previously deemed possible. However, the physiology of the human lung is not adapted to deep breath-hold diving which is associated with a substantial increase in risk of serious neurological injury with increasing depth (Figure 3), rather than actually reaching depth limits defined by individual lung volumes. Recent evidence is increasing that there is significant decompression stress even from single deep breath-hold dives, and it seems that possible adverse effects of pulmonary N_2_ gas exchange during deep breath-hold dives have been largely underestimated in the past. Narcosis is adding to the risk of injury, and its mechanisms in breath-hold diving are only incompletely understood.

## Author Contributions

All authors listed have made a substantial, direct and intellectual contribution to the work, and approved it for publication.

## Conflict of Interest

The authors declare that the research was conducted in the absence of any commercial or financial relationships that could be construed as a potential conflict of interest.

## Footnotes

1 https://worldrecords.aidainternational.org/NoLimit

2 https://www.guinnessworldrecords.com/world-records/freediving-no-limit-(men)
