# Supplementary figures and images for: Going to Extremes of Lung Physiology–Deep Breath-Hold Diving
Source: Front Physiol. 2021 Jul 9;12:710429. doi: 10.3389/fphys.2021.710429 (PMC8299524; doi:10.3389/fphys.2021.710429)

## Slide 1
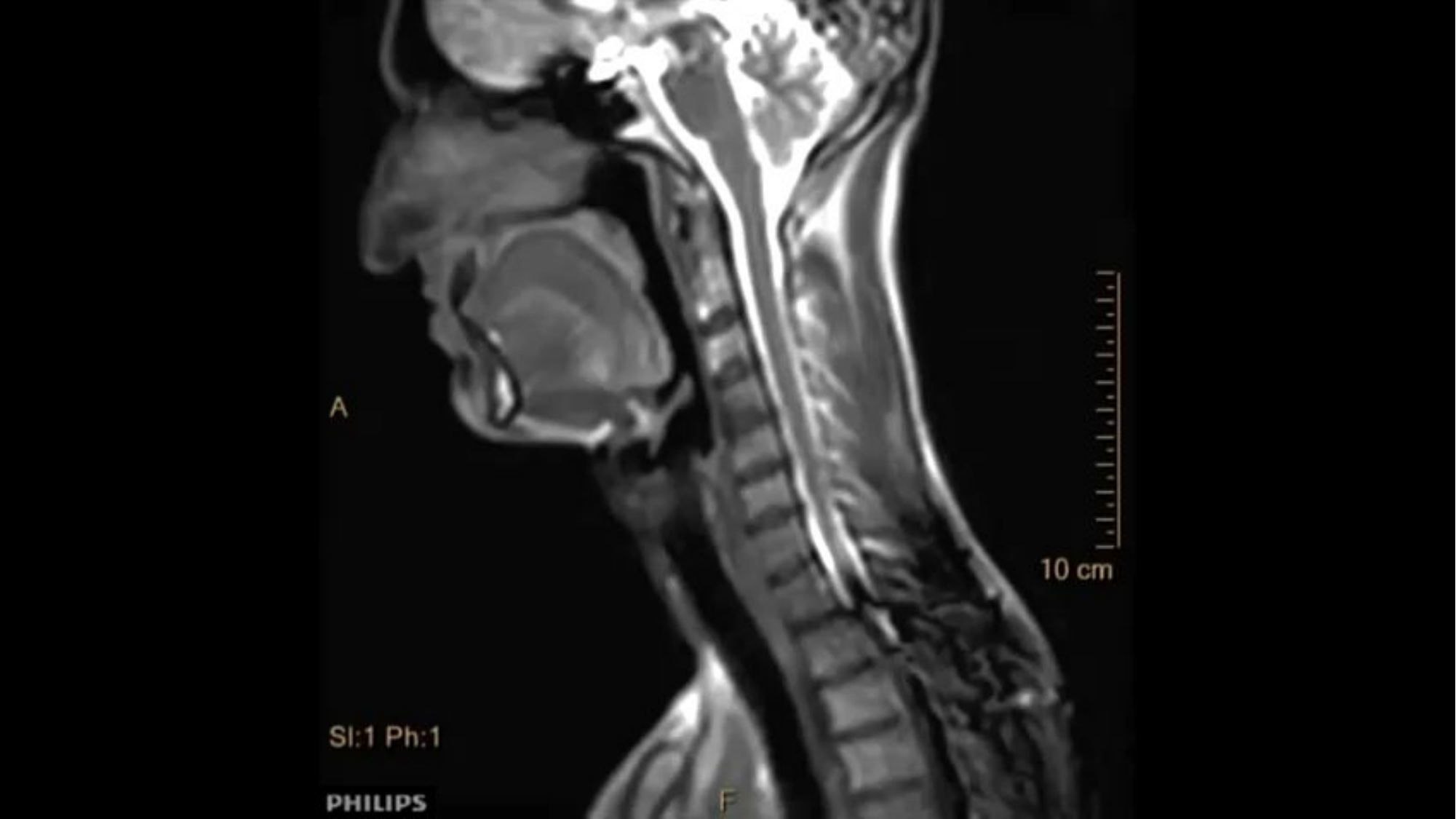

#

Supplement: Supplementary file 1 [file Presentation_1.PPTX]
